# Experimental and Analytical Investigations of the Use of Groove-Epoxy Anchorage System for Shear Strengthening of RC Beams Using CFRP Laminates

**DOI:** 10.3390/ma13194350

**Published:** 2020-09-30

**Authors:** Khalid Mohamed, Jamal A. Abdalla, Rami A. Hawileh

**Affiliations:** 1 Formerly Graduate Student, American University of Sharjah; Sharjah P. O. Box 26666, UAE; b00066746@alumni.aus.edu; 2 Civil Engineering, American University of Sharjah, Sharjah P. O. Box 26666, UAE; rhaweeleh@aus.edu

**Keywords:** carbon fiber reinforced polymer (CFRP), externally bonded reinforcement (EBR), groove-epoxy, shear strengthening, RC beams, anchorage systems, EBR on grooves (EBROG)

## Abstract

Reinforced concrete (RC) beams strengthened in shear with carbon fiber reinforced polymer (CFRP) laminates as externally bonded reinforcement (EBR) usually fail due to debonding. This paper presents an experimental and analytical investigation on the use of groove-epoxy as an anchorage system for CFRP plates and sheets bonded on both sides of shear deficient RC beams. The aim of this study is to assess the effectiveness of using groove-epoxy in enhancing the shear capacity of RC beams. Nine rectangular RC beams were strengthened with CFRP plates and sheets with groove-epoxy anchorage systems of different groove widths and tested under four point bending. It is observed that the RC beams strengthened with the groove-epoxy anchorage system showed an increase in the shear-strength over the unstrengthened control beam up to 112 and 141% for plates and sheets, respectively. Also, the increase of shear-strength contribution of the groove-epoxy system to that of CFRP without grooves ranged between 30–190% for CFRP plates and between 40–100% for CFRP sheets. Generally, the contributions of groove-epoxy on shear-strength decreased with the increase of groove width. Moreover, shear strength prediction models, based on modifications of the ACI440.2R-17 shear model, were developed by incorporating groove factors as a modifier to the FRP shear-strength contribution. The developed models predicted the experimental shear-strength of the tested RC beams with a good level of accuracy, with an average mean absolute percent error (MAPE) = 3.31% and 6.68%, normalized mean square error (NMSE) = 0.072, 0.523, and coefficient of determination R^2^ = 0.964, 0.691, for plates and sheets, respectively.

## 1. Introduction and Background

Reinforced concrete (RC) beams strengthened in shear with carbon fiber reinforced polymer (CFRP) laminates as externally bonded reinforcement (EBR) usually fail due to premature debonding. This usually takes place well before the CFRP utilizes its full tensile strength. To delay or prevent premature deboning, several anchorage techniques have been suggested and employed. The rehabilitation of deteriorated old structures such as buildings, bridges, parking structures, marine structures has become a major issue during the last four decades. This issue has been addressed by many researchers to develop methods and techniques to strengthen, repair, and retrofit such structures.

Strengthening of the deteriorated structures, using externally bonded reinforcement (EBR) materials became one of the most widely used strengthening methods as given by several comprehensive reviews and surveys [1,2,3,4,5,6]. During the last three decades, carbon fiber reinforced polymer (CFRP) composites have become the material of choice that has been used widely for flexure and shear strengthening in the construction industry. This is because it has many advantages, especially in its physical and chemical properties, such as high tensile strength, light weight, and high resistance to corrosion [7,8,9,10,11,12,13,14].

Prior to CFRP materials, other materials have been used in shear and flexural strengthening applications such as steel [15,16,17,18,19,20]. Recently aluminum alloys have been used as externally bonded reinforcement material for both shear and flexure [21,22,23,24,25,26,27,28,29]. Beams are one of major elements in buildings, bridges, and other structures and they can fail in flexure or shear. When strengthening RC beams against shear with CFRP, the fibers do not reach their ultimate strength and mostly debonding of the CFRP happens prematurely. To increase the utilization of the CFRP strength, end anchorage become necessary, especially when the length of the CFRP is limited and the bonded length after a critical section is not enough to reach the ultimate strength of the CFRP laminates. In addition, the problem of premature peeling is also a concern, especially when strengthening a T-section beam because the shear strengthening is only located on the web of the member and the CFRP laminates may end below the position of the neutral axis. As shear strengthening, CFRP has been used extensively as an EBR without anchorage which resulted in premature debonding. Accordingly, many anchorage systems have been developed and used in order to solve the problem of debonding of CFRP sheets or plates by anchoring them to the stem of the beam [30,31,32,33,34,35,36,37,38,39]. Mohee et al. [31] and Kalfat et al. [32] presented a detailed review of use of anchors in general in externally bonded reinforcement using FRP. Mhanna, et al. [35] provided experimental results of shear strengthening of reinforced concrete beams using CFRP wraps. Their experimental test results showed that the beam strengthened by U-Wrapped CFRP sheets increased the shear strength of the tested beams significantly. Mohammed et al. [36] carried an experimental investigation for on using bore-epoxy anchorage system to delay debonding of CFRP plates strengthened concrete beams. They concluded that the use of bore-epoxy delays the debonding of CFRP plates and therefore increased the shear capacity of the tested beams. Koutas et al. [37], Kim et al. [38], and Chen et al. [39] used anchors including CFRP U-strips in shear strengthening of reinforced concrete T-beams with FRP. All reported significant increase in shear capacity due to use of anchors. Furthermore, contributions of externally bonded flexural CFRP laminates in the shear strengthening of RC beams have been investigated by several researchers [40,41,42,43]. Shear strengthening of RC beams is usually achieved by externally bonding CFRP sheets or plates to the vertical sides of the beam’s web with different orientation and spacing using epoxy adhesives. In some situations, the sides of the beam might not be accessible for shear strengthening or might be too shallow to develop the strength of the CFRP laminates without anchors. As a result, flexural reinforcement using CFRP sheets or plates, which are attached to the beam’s soffit, has been used to increase the shear capacity of RC beams. Hawileh et al. [40,41] carried out an experimental study to assess the contributions of flexural CFRP sheets on the shear capacity of RC beams, while Nawaz et al. [42] and Saqan et al. [43] conducted experimental investigation to evaluate the contributions of flexural CFRP plates on the shear capacity of RC beams. They concluded that the shear capacity of RC beams strengthened with CFRP sheets as flexural reinforcement increased by about 10–70% over the control unstrengthened specimen and by about 13–138% for those strengthened with CFRP plates as flexural reinforcement. These studies validated the significant contributions of flexural CFRP sheets and CFRP plates on enhancing the shear capacity of strengthened RC beams.

This study focused on the use of anchorage system in shear strengthening of shear deficient beams using CFRPs plates and sheets as externally bonded reinforcements. Different variables of the anchorage system used have been investigated. The anchorage system investigated in this study is called externally bonded reinforcement on grooves (EBROG) [44,45,46]. The significance of this research is to explore the effect of this type of anchorage system in increasing the shear capacity of RC beams.

Grooving method (GM) includes digging grooves into tension face of concrete beams and filling them with epoxy resin then bonding the CFRP sheets or plates on top of the filled grooves for flexure reinforcement. Mostofinejad et al. [45] applied the EBROG on a set of small-scale beams, where some were strengthened against shear with FRP strips with EBR only and some with EBR on groove (EBROG) method. They found that the beams strengthened with EBR method only showed an increase in the load capacity of 10–13% over the unstrengthened control beam and they failed by FRP debonding, while beams strengthened with EBROG method showed an increase in the load capacity of 17–23% over the unstrengthened control beam and they failed in flexure. A follow up study has been carried out by Mostofinejad et al. [46] using EBROG method for shear strengthening of small RC rectangular beams using CFRP sheets. They concluded that strengthening with CFRP sheets using the EBROG method resulted in a 23% increase in the flexural strength of the beams over the control beam. Moreover, premature debonding has been eliminated and the failure mode has changed from shear to flexural failure with a ductile behavior. Aiming for a comparison investigation for the bond strength between FRP and concrete with externally bonded reinforcement (EBR) and externally bonded reinforcement on grooves EBROG techniques, Hosseini et al. [44] carried out a single shear bond test on prism specimens. They concluded that the EBROG method on prisms has increased the load capacity by 55.5% when compared with conventional EBR method. Other studies were carried out to assess the bond strength of CFRP sheets and strips using EBROG [47,48,49,50]. Mostofinejad et al. [51,52] used another method called externally bonded reinforcement in grooves (EBRIG) for strengthening concrete beams against shear where the difference is that here the grooves are in direct contact with the fibers by inserting the fibers into the grooves. The authors compared EBRIG with the conventional EBR method where they used two-sided, U-shape, and fully wrapped FRP strips for strengthening. They concluded that the EBRIG method combined with the full wrapping has the best performance among all the other with an increase in load capacity by 148% over the control beam, in addition, they noticed that the EBRIG method has changed the failure mode from a brittle shear failure to flexural failure.

Contrary to the previous investigations, this study proposes applying externally bonded reinforcement on grooves on large scale rectangular concrete beams deficient in shear and the mechanism will referred to as groove-epoxy anchorage system. Therefore, the objectives of this study are to: (1) investigate experimentally the effect of using two sided CFRPs sheets and plates on shear strengthening of shear deficient RC beams; (2) examine the effect of using groove-epoxy anchorage systems on the bond between the CFRP laminates and concrete surfaces, tensile strain on CFRP laminates, and beam shear capacity; (3) study the different modes of failure of the strengthened beams when subjected to four points bending; (4) develop analytical models to predict the shear strength of the tested specimens strengthened with CFRP using groove-epoxy anchorage system; (5) use ACI 440.2R-17 provisions to calculate the shear strength of the strengthened RC beams and compare them with the experimental results and with the values predicted by the developed models. The statistical measures such as mean absolute percent error (MAPE), normalized mean square error (NMSE), and coefficient of determination (R2) were used to measure the accuracy of predictions for the developed models compared to experimental values.

## 2. Materials and Methods

A total of nine rectangular RC beams were prepared and tested under four points bending, and they are designed and reinforced in such a way that they have sufficient flexural capacity and fail in shear.

### 2.1. Specimen Details and Test Matrix

#### 2.1.1. Specimen Details

**Figure 1:**
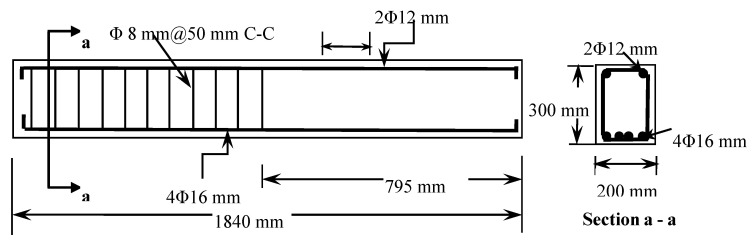
Longitudinal and cross-section details of the beam specimen.

Figure 1 shows the longitudinal section of the beam with reinforcement details. Stirrups, as shear reinforcement, were provided on one side of the beam while the other side was not reinforced with stirrups and therefore it is shear deficient. As shown in Figure 1, the beam specimen dimensions are 1840 × 200 × 300 mm. Figure 1 also shows the cross section of the beam with reinforcement details. As shown, the beams were reinforced in flexure with 4Ø16 mm bars in the tension zone located at 259 mm from the top of the beam. In the compression zone, the beam specimen is reinforced with 2Ø12 mm bars. Each beam was internally reinforced against shear using stirrups of Ø8 at 50 mm center to center as shown in Figure 1.

#### 2.1.2. Specimens Designation and Test Matrix

**Figure 2:**
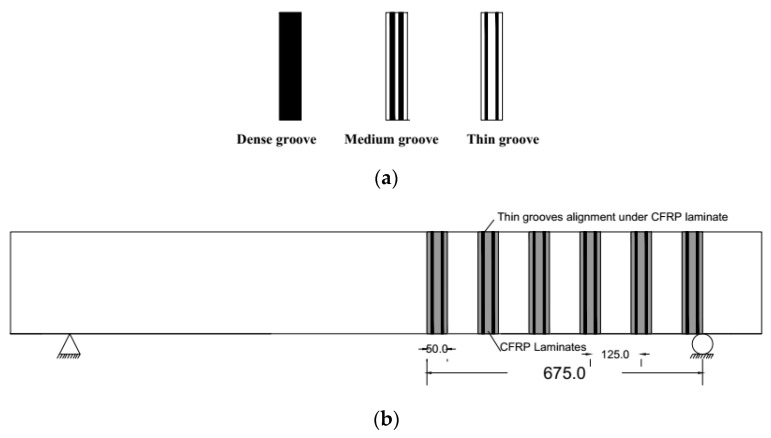
CFRP laminates and grooves alignment (all dimensions are in mm); (**a**) all groove types; (**b**) grooves on beam stem.

As indicated, the nine beams were designed and cast in such a way that the shear failure controls when tested under four points bending. The beams were strengthened using CFRP sheets and plates with two-sided wrapping. Two beams were strengthened using EBR method with CFRP sheets and plates and were designated as S-EBR and P-EBR, respectively. Three beams were strengthened with CFRP sheets using groove-epoxy system with thin, medium, and dense grooves (Figure 2a) and were designated as SGT, SGM, and SGD, respectively where SGT indicates CRFP sheet on thin groove, SGM indicates CFRP sheet on medium groove, and SGD indicates CFRP sheet on dense groove. Similarly, three other beams were strengthened with CFRP plates using groove-epoxy system with thin, medium, and dense grooves and were designated as PGT, PGM, and PGD, respectively where SGT indicates CRFP plate on thin groove, SGM indicates CFRP plate on medium groove and SGD indicates CFRP plate on dense groove. Figure 2b shows a sample beam strengthened with thin groove-epoxy system, two grooves of 10 mm in depth and 5 mm in width were engraved and these two grooves were placed under each of CFRP laminate. Similar procedures were followed for medium (10 mm) and dense (40 mm) grooves.

Table 1 shows the details of the test matrix. It includes a control beam which is unstrengthened and eight strengthened beams. Two of them with EBR and the remaining six with thin (5 mm), medium (10 mm) and dense (40 mm) groove-epoxy anchorage system using plates and sheets of 50 mm in width. The CFRP sheets and plates were spaced at 125 mm with two-sided wrapping as shown in Figure 2b.

### 2.2. Material Properties

#### 2.2.1. Concrete

**Figure 3:**
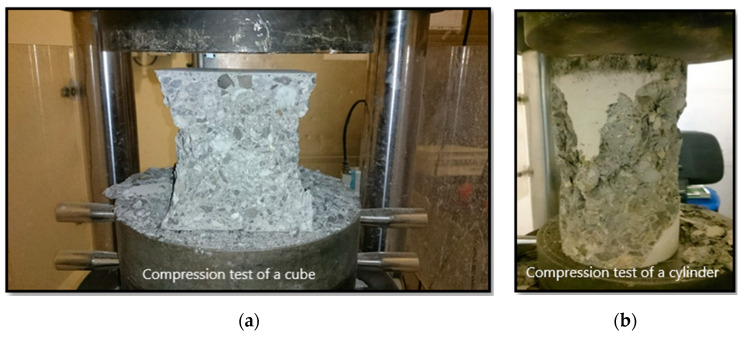
Failure modes of concrete cube and concrete cylinders failure: (**a**) Concrete cube failure mode; (**b**) Concrete cylinder failure mode.

All beams were cast using normal weight concrete mixes of comparable compressive strengths. Three cubes and three cylinders were prepared for each beam and were tested after 28 days. Figure 3a shows mode of failure of for cubes and Figure 3b show mode of failure for cylinders. Several cubes and cylinders were tested and their average cube strength of f_cu_ = 54.4 MPa and average cylinder strength of f_c_ = 41.0 MPa. No study of the microstructural changes was performed since only the final compressive strength of concrete was the main objective.

#### 2.2.2. Steel

Three specimens of steel bars, conforming to ASTM 615, have been tested at a loading rate of 10 mm/min using a universal testing machine (UTM). Three steel bar specimens were tested, and their average yield strength and modulus of elasticity were 590.4 MPa and 199.9 MP, respectively.

#### 2.2.3. CFRP and Epoxy

Sheets are flexible woven FRP filaments or fibres like fabrics and they can be unidirectional or bidirectional. The sheets used in this investigation are unidirectional. Plates are solid laminate of FRP that are made from FRP filaments under temperature and pressure to form a solid material. Table 2 shows the mechanical properties of the CFRP sheets and plates used in this study as stated by the manufacture. CFRP sheets and plates were bonded externally to the RC beams using epoxy adhesive MapeWrap 31 and Adesilex PG2, respectively [53]. Adesilex PG2 is a two-component product based on epoxy resins, selected fine-grain aggregates, and special additives and it hardens in 5 hours’ time without shrinkage. The mechanical properties of the Adesilex PG2 epoxy are also given in Table 2 [53]. MapeWrap 31 is used for the impregnation of sheets/fabrics, for strengthening RC members. MapeWrap 31 is a gelatinous, solvent-free, epoxy resin-based adhesive that is made of two pre-measured components—resin component and hardener component that must be mixed together before use [53]. Table 2 also shows the mechanical properties of MapeWrap 31.

### 2.3. Specimen Preparation

**Figure 4:**
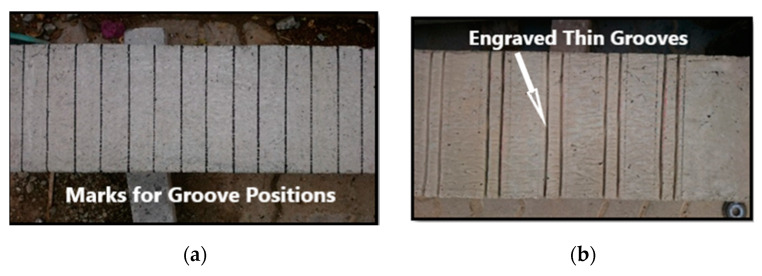

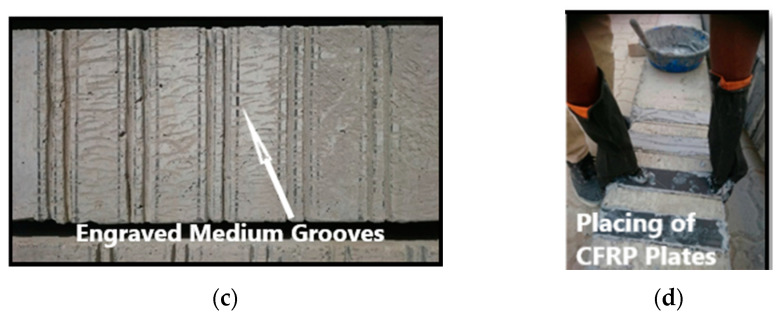
Specimen preparation: (**a**) Marking grooves position; (**b**) Engraving thin grooves; (**c**) Engraving medium grooves; (**d**) Placing CFRP plates and sheets.

For the beams strengthened with EBR the beam surface was cleaned with a brush, place where the CFRP laminates will be attached was marked, a concrete grinder machine was used to remove the thin layer of weak concrete on the sides of the beams to make a rough surface for bonding the CFRP sheet and plates, then an air blower was used to remove all the dust. The wet layup method was then used for bonding the CFRP sheet and plates into the prepared concrete surface by applying a film of an appropriate resin (Mapewrap 31 and Adesllesx PG2) before laying the FRP. The fibers were finally saturated by applying enough resin. Excess resin was removed from the surface to prevent its adverse effect on ultimate rupture strength. For beams strengthened with groove-epoxy anchorage system, the thin, medium, and dense grooves were marked and engraved only on the beam’s sides (beam stem) using the grinder (Figure 4a–c). The grooves were filled with epoxy adhesive and then an epoxy layer of approximately 2 mm was used. Finally, the CFRP sheet or plate was attached on top of the epoxy layer (beam’s sides) and were impregnated with another layer of epoxy adhesive (Figure 4d). The specimens were left to cure for 7 days waiting for the epoxy to gain its full strength prior to testing.

### 2.4. Test Setup and Instrumentations

**Figure 5:**
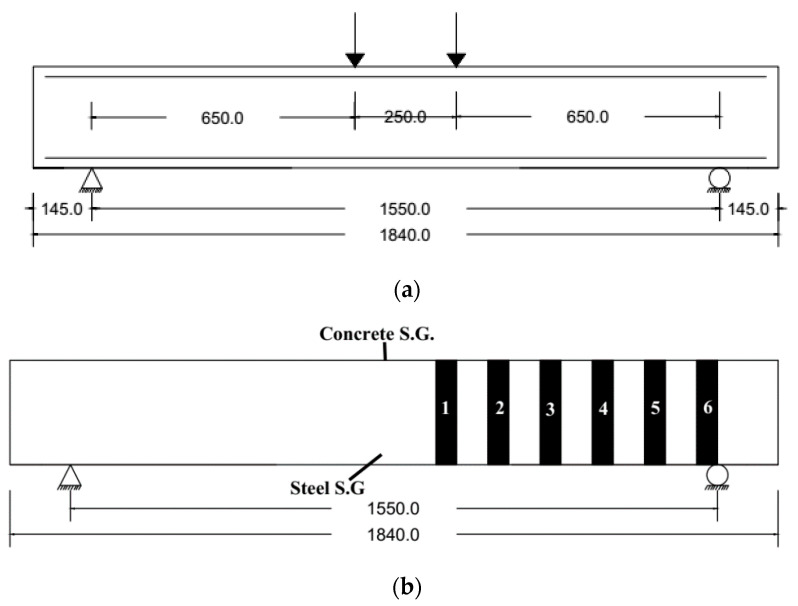
Test setup and instrumentations (all dimensions are in mm): (**a**) Loading setup; (**b**) Strain gauges location.

**Figure 6:**
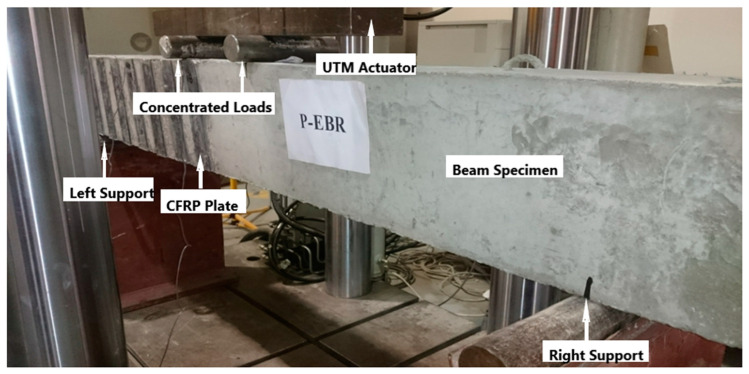
Test specimen mounted in the universal testing machine.

Figure 5a displays the test setup with a 250 mm distance between the load points. The beam clear test span was 1550 mm with a shear span of 650 mm and shear span to depth ratio (a/d) of 2.51 as shown in Figure 5a. Figure 5b shows the locations of strain gauges on concrete, steel and CFRP sheets and plates. All beams were loaded monotonically using a digitally controlled INSTRON 8806 Universal Testing Machine (UTM) as in Figure 6. The UTM has a capacity of 2500 kN and the member were tested under a load control rate of 10 kN/min. All experiments were conducted at room temperature and at room humidity, consistently.

## 3. Experimental Results and Discussion

### 3.1. Summary of Experimental Results

This section includes the results of the experimental program conducted in this investigation. As indicated, all the samples were tested under four point bending and the applied force and corresponding deflection at the beams’ mid span were recorded and plotted, strain graphs of the data recorded from strain gauges (concrete, steel and CFRP) are shown for each beam. In addition, captions of the failed beam specimens are provided to demonstrate the modes of failure. Table 3 presents summary of the experimental results in terms of ultimate attained load, corresponding deflection at mid span and the percentage increase over the control beam with the associated failure mode for each beam specimen.

### 3.2. Load Deflection Relationships and Modes of Failure

#### 3.2.1. Specimens C, S-EBR and P-EBR

**Figure 7:**
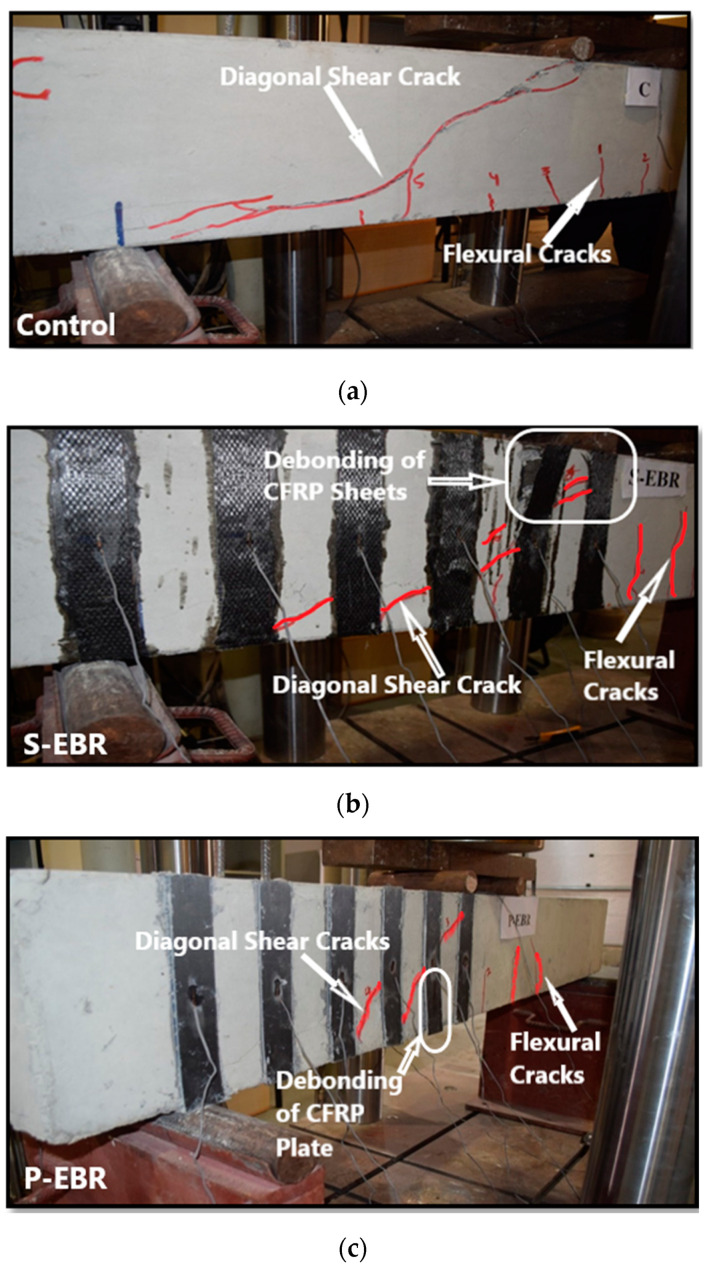
Failure modes: (**a**) Diagonal shear crack on the control beam; (**b**) debonding of CFRP sheets in S-EBR; (**c**) debonding of CFRP plates in P-EBR.

The control beam failed in shear as expected with a capacity of 116.96 kN. As shown in Figure 7a, the shear crack took place in the half of the unreinforced side of the beam with shear reinforcement (stirrups), it started from the point of load application and propagated to the support of the beam at an angle of approximately 45 degrees. This failure mode is expected since the a/d is 2.51, which is between 2.5 and 5.

The S-EBR beam was strengthened in shear with CFRP sheets using EBR method. This beam specimen failed in shear with a diagonal tension crack experiencing higher load and displacement compared to the control beam. The ultimate load sustained by S-EBR was 190.04 kN, which is 62.5% higher than the control beam, with a corresponding maximum deflection of 8.85 mm. At the onset of failure, diagonal cracks were formed and passed under CFRP sheets number 2 and 3 and sudden debonding of these sheets took place as shown in Figure 7b. This occurred mainly due to the weak interface between the CFRP sheets and concrete beam.

The P-EBR beam was strengthened in shear with CFRP plates using EBR method. This beam specimen failed in shear with a diagonal tension crack experiencing higher load and displacement compared to the control beam. The ultimate load was 162.90 kN, which is 39.3% higher than the control beam, with an associated deflection of 7.10 mm. At the onset of failure, a diagonal crack was formed and passed under CFRP sheets number 2 and 3, then sudden debonding of CFRP plates happened and that is also due to the weak interface between the CFRP plates and concrete beam as shown in Figure 7c.

#### 3.2.2. Specimens PGT, PGM and PGD

**Figure 8:**
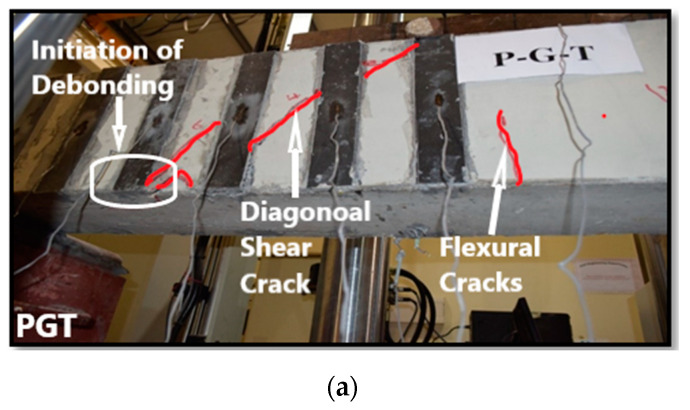

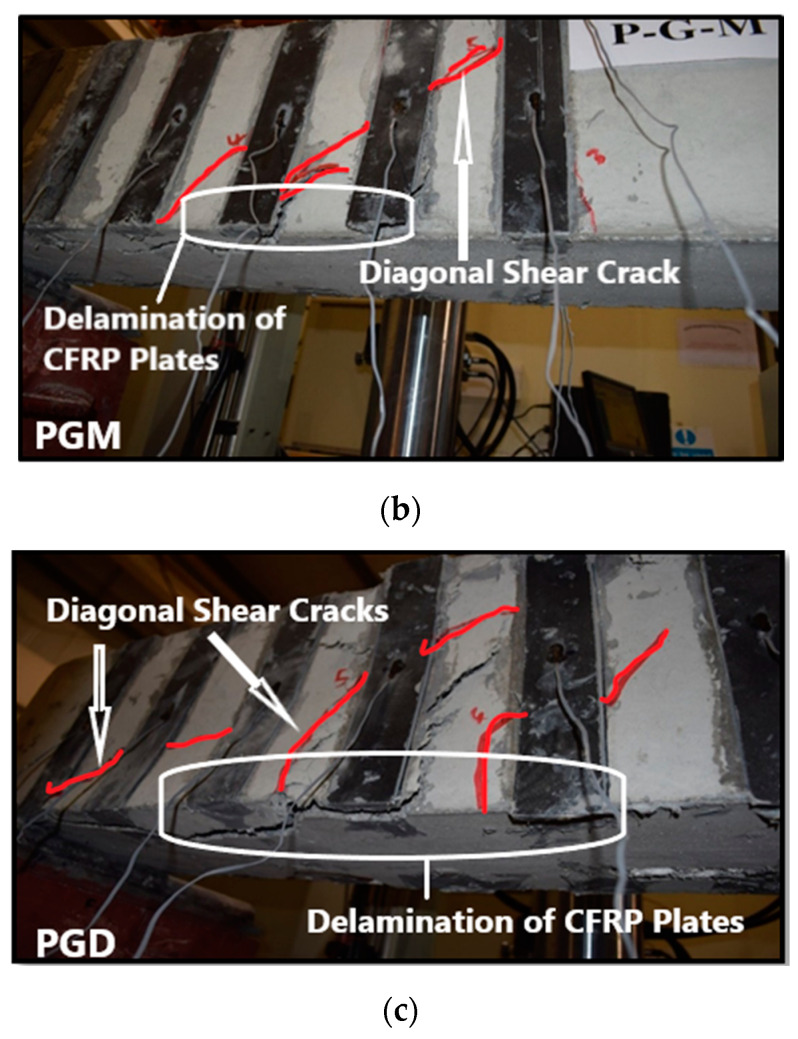
Failure modes: (**a**) PGT beam; (**b**) PGM beam; (**c**) PGD beam.

The PGT beam was strengthened in shear with CFRP plates using groove-epoxy anchorage with two thin grooves. It failed in shear with a diagonal tension crack experiencing higher load and displacement compared to control beams. The ultimate load was 241.68 kN with a deflection of 8.77 mm, which is 106.63% higher than the control beam as shown in Table 3. At the onset of failure, diagonal crack passed under CFRP plate number 2 and 3 and delamination of CFRP plate number 2 happened, as shown in Figure 8a. The PGM beam was strengthened in shear with CFRP plates using groove-epoxy anchorage with two medium grooves. It failed in shear with a diagonal tension crack experiencing higher load and displacement compared to the control beam. The ultimate load was 248.30 kN with a deflection of 7.79 mm (Table 3), which is 112.29% higher than the control beam. It also showed larger attained load than the PGT sample, which means that the groove width has an effect in delaying debonding. At last, because of the weak interface between the CFRP plates and concrete beam, diagonal crack passed under CFRP plate number 2 and 3 and delamination of CFRP plate number 2 happened as shown in Figure 8b. The PGD beam was strengthened in shear with CFRP plates using groove-epoxy anchorage with one dense groove. It failed in shear with a crack that started from the load point and went through the top of the beam until it reached the third CFRP plate, then it went down diagonally and continued through the bottom until it reached the beam’s edge, experiencing higher load and displacement compared to the control beam. The ultimate load was 176.30 kN with an associated deflection of 7.72 mm (Figure 8), which is 50.74% only higher than the control beam but less than PGT and PGM. Thus, two thin or medium groves performs better than one dense groove in delaying plate debonding. Eventually, delamination of CFRP plates 2 and 3 happened, as shown in Figure 8c.

**Figure 9:**
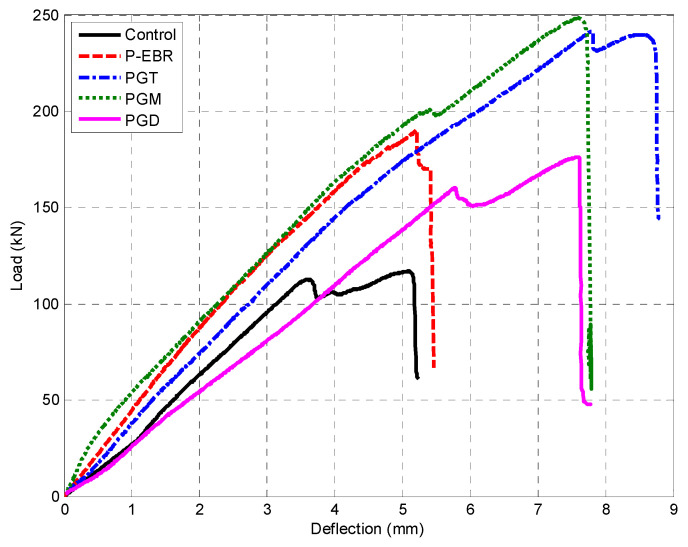
Load deflection curves for PGT, PGM, and PGD beam.

Figure 9 shows the load-deflection relationship for the beams with groove-epoxy anchorage systems using CFRP plates (PGT, PGM and PGD). The thin and medium grooves achieved the maximum load and slightly more deflection among the three specimens that were strengthened with groove-epoxy, as shown in Figure 9. All the beams that used groove-epoxy anchorage systems consistently showed higher strength than that of the conventional EBR strengthening method.

#### 3.2.3. Specimens SGT, SGM and SGD

**Figure 10:**
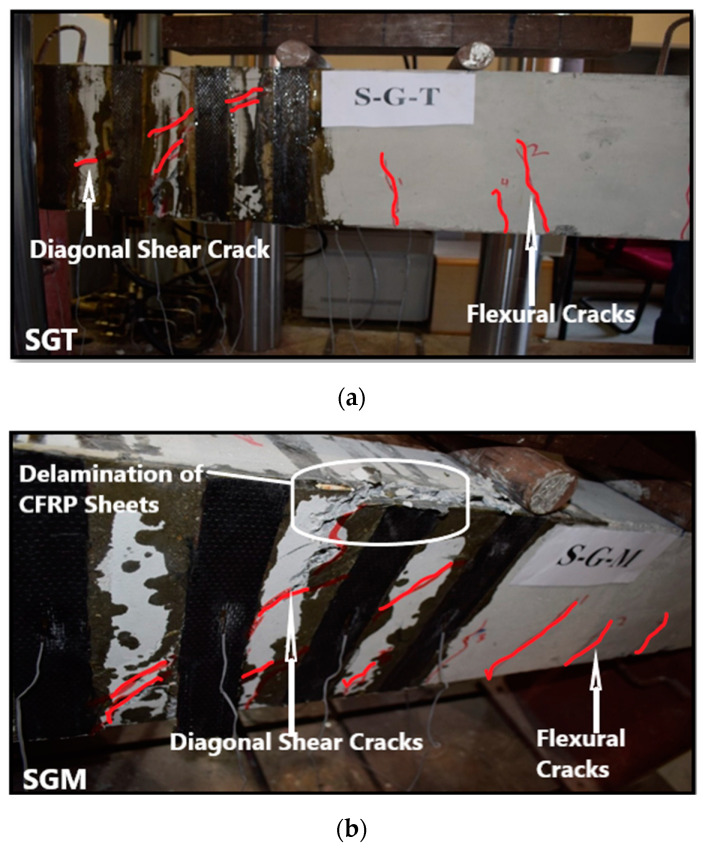

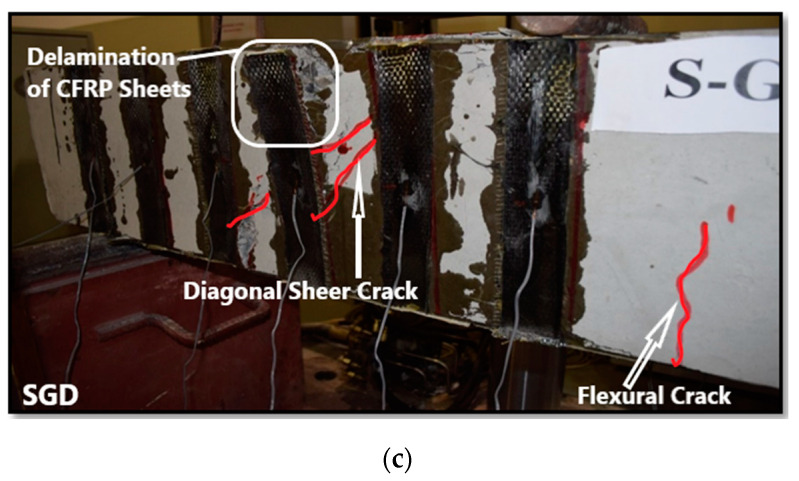
Failure modes: (**a**) SGT beam; (**b**) SGM beam; (**c**) SGD beam.

**Figure 11:**
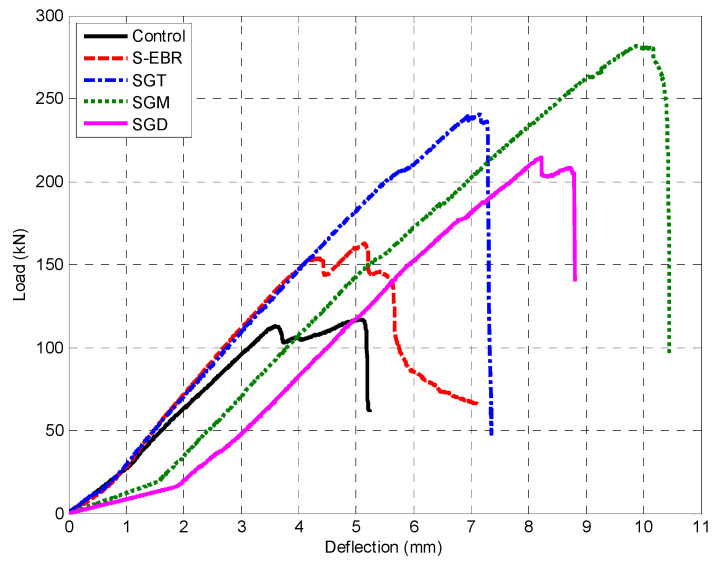
Load deflection curves for SGT, SGM, and SGD beam.

The SGT beam was strengthened in shear with CFRP sheets using groove-epoxy anchorage with two thin grooves. It failed in shear with a crack started from the load point and going down until the third CFRP sheet and then continued through the beam bottom until it reached the edge experiencing higher load and displacement compared to control beams. The ultimate load was 240.60 kN with deflection of 7.35 mm (Table 3) which is 105.71% higher than the control beam. It’s observed that the thin groove-epoxy anchors helped in delaying the debonding of CFRP sheets. At last, diagonal crack passed under CFRP plate numbers 2 and 3 and delamination of CFRP sheet number 2 happened as in Figure 10a. The SGM beam was strengthened in shear with CFRP sheets using groove-epoxy anchorage with two medium grooves. It failed in shear with a crack started from the load point and going down after the second sheet and started going down diagonally until the third CFRP sheet then continues through the beam bottom until it reached the edge experiencing higher load and displacement compared to control beams. The ultimate load was 281.89 kN with deflection of 10.93 mm (Table 3) which is 141.01% higher than the control beam. It can be noticed that the medium groove-epoxy anchors helped in considerably delaying the debonding of CFRP sheets. Ultimately, delamination of the second and third CFRP sheet happened, as shown in Figure 10b. The SGD beam was strengthened in shear with CFRP sheets using groove-epoxy anchorage with one dense groove. It failed in shear with a crack that started from the load point and went through the top of the beam causing the first and third CFRP sheets to delaminate, then it passed diagonally to the bottom, and continued through the bottom until it reached the beam’s edge experiencing higher load and displacement compared to the control beam. The ultimate attained load was 214.56 kN with a deflection of 8.85 mm, as depicted in Figure 11, which is 83.45% higher than the control beam. It can be noticed that the dense groove-epoxy anchors helped in delaying the debonding of CFRP sheets. At last, some sheets were delaminated as shown in Figure 10c.

Figure 11 shows load and deflection relationship for the beams with groove-epoxy anchorage strengthening method using CFRP sheets (SGT, SGM and SGD). The sheets with medium grooves achieved the highest attained load along with the highest associated deflection among the three specimens, which proved that there is an optimum size for the groove’s effectiveness. Nevertheless, all the beams that used groove-epoxy anchorage system achieved higher strength compared to the beam with conventional EBR method.

**Figure 12:**
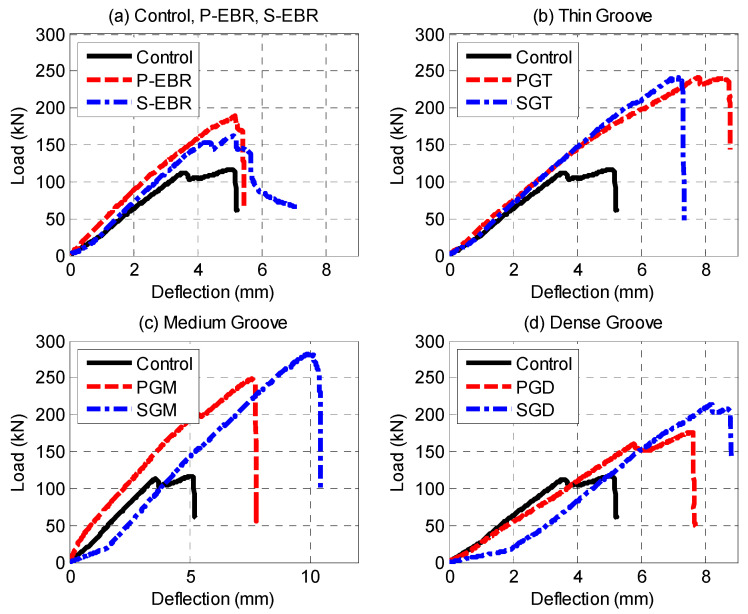
Load-deflection curves: (**a**) Control, P-EBR, S-EBR; (**b**) Thin grooves; (**c**) Medium grooves; (**d**) Dense grooves.

Figure 12 shows the effect of groove width on the shear capacity of RC beams. Overall, the groove-epoxy anchorage system with CFRP sheets on medium groove size shows the highest ultimate shear load capacity and highest deflection compared to all tested specimens as shown in Figure 12c. It is also observed from Figure 12b that for the beams strengthened with EBR on thin grooves, the stiffness of beams strengthened with plates and sheets is the same and the ultimate shear capacity is also quite similar with a slight difference in the ultimate displacement. The medium grooves showed a larger shear capacity than the thin and the dense grooves. This suggested that there is an optimum groove width for the given groove depth that is close to the medium width. As the width of grooves increases (dense grooves) the case resembles an EBR with deep epoxy only, while as the thickness of grooves decreases (thin grooves), the effectiveness of grooves diminishes due to the decrease in groove width that is in contact with the plate or the sheet, which results in a case that resembles an EBR.

In summary, all specimens failed in shear with a major diagonal shear crack followed by CFRP sheets or plates debonding or delamination and some flexural cracks or flexural-shear cracks. Crushing of concrete at the point of application of the load took place for some specimens. Although all failures were brittle shear failure, however, the use of groove epoxy delayed the debonding of CFRP laminates and therefore increased the shear capacity of the strengthened beams. The appearance of small flexural and flexural-shear cracks had little contribution, if any, to the brittle shear failure of the tested beams. At the onset of failure, diagonal shear cracks usually pass under the CFRP plates or sheets. Even for the debonding failure mode, it took place at a higher strain level, but still below the CFRP rupture’s strain.

## 4. Load Strain Relationships

Table 4 display the recorded strain values for concrete, steel, and CFRP sheets and plates for all the beams during testing.

**Figure 13:**
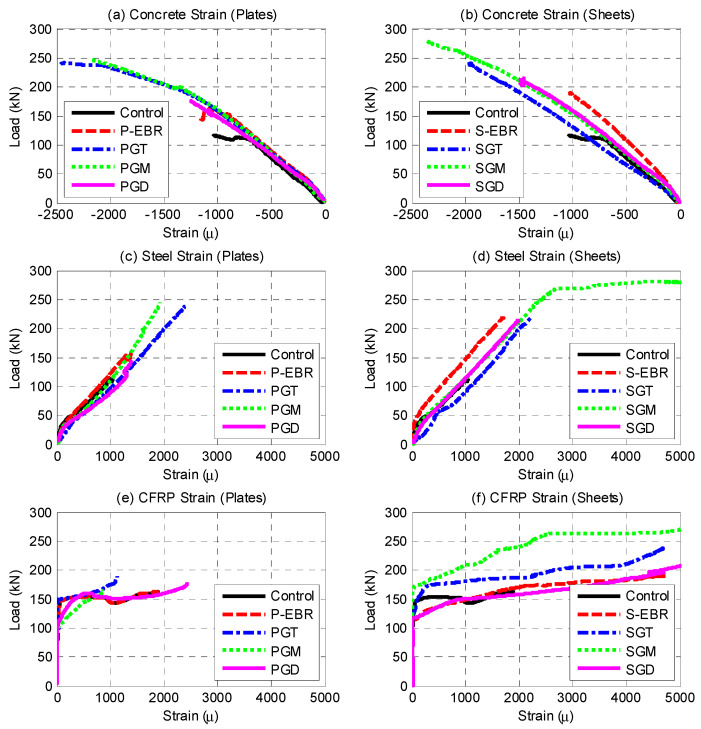
Load-strain relationships for concrete, steel and CFRP laminates: (**a**) Concrete strain (Plates); (**b**) Concrete strain (Sheets); (**c**) Steel strain (Plates); (**d**) Steel strain (Sheets); (**e**) CFRP strain (Plates); (**f**) CFRP strain (Sheets).

Figure 13a,b show the load versus microstrain response curves for the concrete of all tested samples. The maximum strain reached by concrete is less than the crushing value of concrete. Figure 13c,d show the load-strain for steel in the RC beams strengthened with plates and sheets, respectively. It is clear that the maximum strain reached by the flexural steel reinforcement is less than the yielding value for steel, confirming that the steel did not yield.

As previously indicated, six strain gauges were attached to the CFRP sheets to record the strain while applying the load in each of the beams P-EBR, PGT, PGM, and PGD, and were attached at the center of every CFRP sheet. They showed a maximum microstrain of 4740.4, 4697.2, 15,834.0, and 7800.9 which represents 23.7%, 23.5%, 79.2%, and 39.0% of the ultimate strain of the CFRP sheets (20,000) as shown in Figure 13e. These strain values proved that the groove-epoxy system increased the strain in the CFRP sheets. Six strain gauges were attached to the CFRP plates to record the strain while applying the load in each of the beams S-EBR, SGT, SGM and SGD, and were attached at the center of every CFRP plate. They showed maximum microstrain values of 1895.8, 1133.7, 873.5, and 2456.5 which represents 9.5%, 5.7%, 4.4%, and 12.3% of the ultimate strain of the CFRP plates (20,000) as shown in Figure 13f. These strain values proved that groove-epoxy system increased the strain in the CFRP plates as well.

## 5. Models for Prediction of Shear-Strength of Groove-Epoxy Anchorage

### 5.1. ACI440.2R Prediction of Shear-Strength Capacity of RC Beams

ACI 440.2R-17 [54] presents guidelines for using FRP in flexure and shear applications. These guidelines are based on a limit-states-design that sets limits on both serviceability and ultimate limit states. The deboning failure mode (brittle failure mode) takes place at a strain level below the FRP rupture strain. The ACI specified a maximum attainable strain of 0.4% in the FRP wraps which have been adopted from Khalifa et al. [55] who proposed a maximum strain limit of 0.4% to maintain the shear integrity of the concrete and prevent loss of aggregate interlock. For completely wrapped elements: the effective strain level in FRP reinforcement attained at failure ε_fe_ = 0.004 ≤ 0.75ε_fu_. However, for U-wraps and side applications the ACI guidelines introduce a bond-reduction coefficient K_v_, as such FRP applications are susceptible to the debonding failure mode. The bond reduction coefficient K_v_ that was experimentally derived is dependent upon several factors, including concrete strength, type of wrapping scheme used, and stiffness of the FRP laminate. The following equations were used in predicting the shear strength of the tested specimens.

(1) $$\phi V_{n}=\phi \left(V_{c}+V_{s}+\Psi V_{f}\right)$$

where *V_c_*, *V_s_*, and *V_f_* are the shear strength contribution of concrete, stirrups, and FRP, respectively.

### 5.2. Proposed Model for Predicting Shear Capacity of CFRP Plate on Groove-Epoxy

From the experimental results two empirical shear capacity prediction models have been developed, one for CFRP plates and the other for CFRP sheets mounted on groove-epoxy system. The models are based on a groove factor *G_FP_* for plates or *G_FS_* for sheets. This factor incorporates the shear-strength contribution due to the use of grooves in the shear-strength contribution of the FRP (i.e., *V_f_*) of ACI440.2R-17 [54] shear prediction equation. The groove factors (*G_FP_* or *G_FS_*) are defined as the ratio of the shear-strength contribution of RC beam strengthened with CFRP plates/sheets on groove-epoxy over the shear-strength contribution of CFRP as EBR without groove-epoxy.

**Figure 14:**
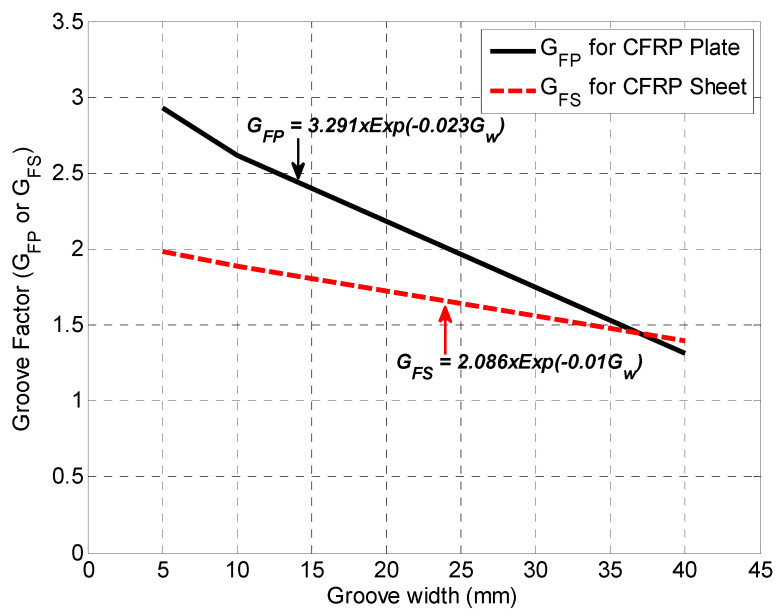
Groove Factors (GFP and GFS) for CFRP plats and sheets on groove-epoxy.

The nominal shear capacity of the RC beam strengthened with CFRP plates on groove-epoxy, including contributions of concrete, steel reinforcement and FRP reinforcement, is given by Equation (2). This is the ACI440.2R-17 [54] equation that has been modified by including a Groove Factor (*G_FP_* or *G_FS_*) to the shear-strength contribution of the FRP. The groove factor *G_FP_* for CFRP plate is given by Equation (3) and its variation with groove width is shown in Figure 14. The average mean absolute percent error (MAPE) for prediction MAPE = 6.04%, normalized mean square error (NMSE) NMSE = 0.072, and coefficient of determination R^2^ = 0.964.

(2) $$V_{n}=V_{c}+V_{s}+G_{FP}\Psi_{f}V_{f}$$

where:

(3) $$G_{FP}=\left\{ \begin{array}{l} 3.291\times Exp(-0.023G_{w})\;\;for\;\;5\;mm\;\leq G_{w}\;\leq 40\;mm\;\\ 1.0\;Otherwise \end{array}\right.\;G_{w}=Groove\;\;\;\;width\;\;\;\;\left(mm\right)$$

### 5.3. Model for Predicting Shear Capacity of CFRP Sheet on Groove-Epoxy

The nominal shear capacity of the RC beam strengthened with CFRP sheets on groove-epoxy is given by Equation (4) and the groove factor for CFRP sheets is given by Equation (5) and also depicted in Figure 14. The average MAPE = 12.79%, NMSE = 0.525 and R^2^ = 0.689.

(4) $$V_{n}=V_{c}+V_{s}+G_{FS}\Psi_{f}V_{f}$$

where:

(5) $$G_{FS}=\left\{ \begin{array}{l} 2.086\times Exp(-0.01G_{w})\;\;for\;\;5\;mm\;\leq G_{w}\;\leq 40\;mm\;\\ 1.0\;Otherwise \end{array}\right.\;G_{w}=Groove\;\;\;\;width\;\;\;\;\left(mm\right)$$

**Figure 15:**
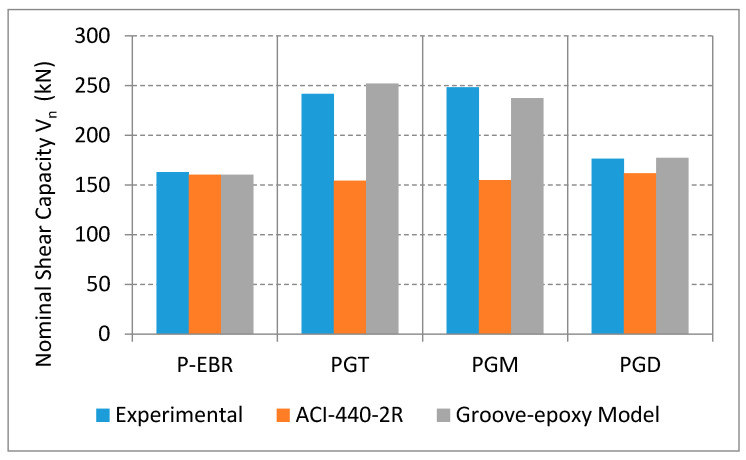
Comparison of measured and predicted shear capacity for RC beams strengthened with CFRP plates on groove-epoxy.

Table 5 illustrates the experimental shear strength (V_exp_) and the theoretical shear strength at different strain levels. ACI equations use ($K_{v}\times \varepsilon_{\mathrm{fu}}$) as a limit for the strain used to predict the shear strength. The shear strength is also calculated using the strain values obtained from the CFRP strain gauges. All these predicted values were less than the experimental ones, therefore, higher strain values were used to calculate the shear strength (3000 and 6000 μstrain). Figure 15 represents the experimental and the model predicted shear strength values for P-EBR, GT, PGM, and PGD beams as well as those using the ACI equation with strain limit. It has been observed that the actual shear strength valued falls between strain values of 3000 to 6000 μstrain, which means that the ACI limit ($K_{v}\times \varepsilon_{\mathrm{fu}}$) needs to be adjusted when using the groove-epoxy anchorage system with CFRP plates.

**Figure 16:**
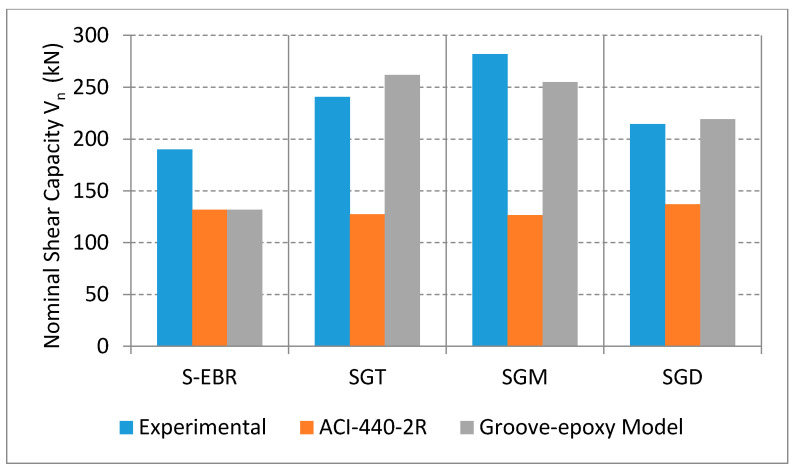
Comparison of measured and predicted shear capacity for RC beams strengthened with CFRP sheets on groove-epoxy.

Table 6 illustrates the experimental, the ACI and the model predicted shear strength values. The ACI value was based on the imposed strain limit of $K_{v}\times \varepsilon_{\mathrm{fu}}$. It is observed that the predicted values were less than the experimental ones, therefore, higher strain values were used to calculate the shear strength (2500 and 4500 μstrain). Figure 16 represents the experimental and the model predicted shear strength values for S-EBR, SGT, SGM, and SGD beams as well as the ACI ones using the ACI equation with the strain limit. It can be deduced that the actual shear strength values falls between strain values of 2500 and 4500 μstrain, which means that the ACI limit ($K_{v}\times \varepsilon_{\mathrm{fu}}$) needs to be increased when using groove-epoxy anchorage system with CFRP sheets. Therefore, the proposed models, with groove-epoxy factors, could be used to predict the shear capacity of the strengthened specimens with a reasonable level of accuracy.

As previously indicated, the groove-epoxy anchorage system, also known as EBROG, has been used mainly for anchoring FRP for flexural strengthening and it was very successful in delaying debonding and therefore improving the flexural capacity of the strengthened RC beam specimens. Very little work had been attempted in using groove-epoxy in anchoring FRP for shear strengthening of RC beams with practical dimensions. Experimental programs that used small-size RC beams (prisms) with dimensions of 70 × 85 × 560 mm [45] and 70 × 85 × 570 mm [46], explored the use of groove-epoxy or ERBOG on anchoring of CFRP sheets for shear strengthening [45,46]. They concluded that EBROG method is a very efficient technique in increasing the shear capacity of the tested small-size beams by up to 23% over unstrengthened control beam for one layer of CFRP sheet. In this study large-size RC beams with dimensions of 200 × 300 × 1840 mm were tested and the increase in shear capacity reached up to 141% over the unstrengthened control beam. It is clear that the results of both experimental investigations showed an increase in the shear capacity of the tested specimens, however, the strengthened specimens and the size effect may have contributed to the variation of percent increase in the shear capacity.

Several researchers have thoroughly investigated the size effect on the shear strength and shear failure mode of RC beams and thus identified several parameters that have different level of influence on the size effect [56,57,58,59,60,61,62,63]. Such parameters included shear-span-to-depth ratio (a/d), beam depth (d), maximum aggregate size, and concrete strength (fc), among others. It was observed that using small size specimens may lead to overestimation of shear strength of beams without stirrups. The beam specimens tested in this investigation did not have stirrups on one side and they all failed in shear with a major diagonal shear crack that propagated until failure. The “a/d” ratio of all tested beams has been kept at a constant value of 2.51 to ensure shallow (slender) beam behavior. Since all beams failed in shear, there is a strong size effect on the experimental values of the shear strength of the tested beams and also on the brittleness of their failure mode. Such size effect should be taken into consideration when comparing the experimentally measured shear strength values of this investigation to those of beams with larger sizes.

## 6. Summary and Conclusions

In this study the problem of early debonding of CFRP sheets and plates has been addressed, when used in shear strengthening application of reinforced concrete beams. It can be concluded from this investigation that:

1. Using the EBR conventional method showed an increase in the shear capacity over the control beam by 39.28% and 62.48% for CFRP plates and sheets strengthened specimens, respectively.
2. When CFRP plates are used, groove-epoxy anchors had increased the shear capacity up to 112.29% over the control beam and 52.42% over the EBR strengthened beam. While CFRP sheet specimens showed an increase of 141.01 and 48.36% over the control and EBR strengthened specimens, respectively.
3. The groove-epoxy anchorage system increased the shear-strength contribution of CFRP without grooves in the range of 30–190% for CFRP plates and 40–100% for CFRP sheets.
4. Generally, the contributions of groove-epoxy on shear-strength decreases with the increase of groove width, with the medium groove specimens showed the best performance among the other groove sizes.
5. All specimens failed in shear with a major diagonal shear crack, followed by CFRP sheets or plates debonding, or delamination and some combination of small flexural and flexural-shear cracks.
6. The groove-epoxy method changed the mode of failure from debonding to delamination for almost all specimens and delayed the early debonding failure of CFRP sheets and plates for others, and consequently increased the beams’ shear strength and maximum deflection.
7. The developed shear-strength prediction models incorporated groove factors as a modifier to the FRP shear-strength contribution of ACI-440 shear equation. The developed models predicted the experimental shear-strength of RC beams with the utilized groove-epoxy systems with a good level of accuracy, with an average MAPE = 3.31% and 6.68%, NMSE = 0.072, 0.523 and coefficient of determination R^2^ = 0.964, 0.691, for plates and sheets, respectively.

## Figures and Tables

**Table 1:** Details of the test matrix.

No	Specimen	FRP Type	Strengthening Technique	Groove Width (mm)	FRP Width (mm)	FRP Spacing S_f_ (mm)
1	C1	—	Control Beam Unstrengthen	—	—	—
2	S-EBR	Sheet	EBR	—	50	125.0
3	SGT	Sheet	Thin Groove-Epoxy Anchor	5	50	125.0
4	SGM	Sheet	Medium Groove-Epoxy Anchor	10	50	125.0
5	SGD	Sheet	Dense Groove-Epoxy Anchor	40	50	125.0
6	P-EBR	Plate	EBR	—	50	125.0
7	PGT	Plate	Thin Groove-Epoxy Anchor	5	50	125.0
8	PGM	Plate	Medium Groove-Epoxy Anchor	10	50	125.0
9	PGD	Plate	Dense Groove-Epoxy Anchor	40	50	125.0

**Table 2:** Mechanical properties of CFRP sheets, plates and epoxy.

Material	Thickness (mm)	Modulus of Elasticity (GPa)	Ultimate Tensile Strength (MPa)	Elongation at Failure (%)	Density (g/cm^3^)
CFRP Sheets	0.17	230	4800	2.0	1.79
CFRP Plates	1.40	170	3100	2.0	1.61
Adesilex PG2	-	6	≥18	-	1.70
Mapewrap 31-	-	≥3	≥40	≥1.6	1.06

**Table 3:** Summary of experimental results.

No	Specimen	Load (kN)	Deflection (mm)	Increase over Control Beam (%)	Increase over EBR Beams (%)	Failure Mode
1	C	116.96	5.24	—	—	Major shear crack
2	P-EBR	162.90	7.10	39.3	—	Major shear crack + CFRP debonding
3	PGT	241.68	8.77	106.6	48.4	Major shear crack + CFRP delamination
4	PGM	248.30	7.79	112.4	52.4	Major shear crack + CFRP delamination
5	PGD	176.30	7.72	50.7	8.2	Major shear crack + CFRP delamination
6	S-EBR	190.04	5.85	62.5	—	Major shear crack + CFRP debonding
7	SGT	240.60	7.35	105.7	26.6	Major shear crack + CFRP delamination
8	SGM	281.89	10.93	141.0	48.3	Major shear crack + CFRP delamination
9	SGD	214.56	8.85	83. 5	12.9	Major shear crack + CFRP delamination

**Table 4:** Summary of experimental results.

No.	Specimen	Maximum Strain in Concrete (μs)	Maximum Strain in Steel (μs)	Maximum Strain in CFRP Laminates (μs)
1	C	1046.0	1044	—
2	P-EBR	1155.8	1390.9	1895.8
3	PGT	3073.1	2388.0	1133.7
4	PGM	2154.2	1930.3	873.5
5	PGD	1248.9	1390.9	2456.5
6	S-EBR	1027.8	1709.9	4740.4
7	SGT	1987.6	2438.9	4697.2
8	SGM	2360.0	5077.7	15,834.0
9	SGD	1498.9	1983.7	7800.9

**Table 5:** Measured and predicted loads for RC beam strengthened with CFRP plates on groove-epoxy.

Designation	Experimental (kN)	ACI-440 Strain Limit (kN)	Groove-Epoxy Model (Equation (2)) (kN)	ACI-440 MAPE (%)	Model MAPE (%)
C	117.0	114.28	—	—	—
P-EBR	162.9	160.4	160.4	1.53	1.53
PGT	241.7	154.321	252.194	36.15	4.35
PGM	248.3	154.892	237.503	37.62	4.36
PGD	176.3	161.797	177.421	8.25	0.61

**Table 6:** Measured and predicted loads for RC beam strengthened with CFRP sheets on groove-epoxy.

Designation	Experimental (kN)	ACI-440 Strain Limit (kN)	Groove-Epoxy Model (Equation (4)) (kN)	ACI-440 MAPE (%)	Groove-Epoxy Model MAPE (%)
C	117.0	114.28	—	—	—
S-EBR	190.04	131.9	131.0	30.59	30.59
SGT	240.62	127.3	261.970	47.10	8.15
SGM	281.89	126.5	254.898	55.12	10.59
SGD	214.57	136.9	219.147	36.20	2.099
